# What to Do with Porcelain

**Published:** 1904-02-15

**Authors:** W. T. Reeves

**Affiliations:** Chicago


### WHAT TO DO WITH PORCELAIN.

BY W. T. REEVES, D.D.S., CHICAGO.

What to do with porcelain—make use of it to fill teeth in all parts of the mouth.

Clinical facts have demonstrated that porcelain is par excellence as a filling material. It has won its place against stubborn conservatism, by hard fought efforts on the part of its advocates.

The fact of the matter is the opposition has been so persistent and long drawn out because the opposers could not do the work. Patients are fast finding out that it is lack of ability and not lack of material that is behind the cry of wolf; they are going to those who use porcelain and can put in porcelain fillings.

Two of the latest excuses or arguments against porcelain came to my attention this past month, namely, that they are always coming out, and that you have to cut away so much good sound tooth structure in order to be able to put in inlays. Who of you would consider the first as a valid reason for not putting on a crown where a crown was indicated? There is an occasional crown that loosens and has to be set over again, you all meet with that in your practice, but who of you would for a moment consider that any ground against crowning or bridging? There is an occasional inlay that loosens and has to be set again. We cannot tell why this crown or inlay should loosen and have to be set again when so many others never loosen. We mix the cement in all cases as nearly the same as we can make one mix like another, and yet one loosens and the others do not. In the case of the inlay it is just as likely to be one that is not subject to occlusal stress as one that is. A patient presenting himself with an inlay to be set does not bother me any more than you are bothered by a patient coming with a crown that has loosened.

The second argument, that you have to cut away so much good sound tooth structure in order to be able to put in inlays is almost the limit, but it is very effective, for if there is one thing more than another that patients dislike, it is to lose any remnant of tooth that they may have left, and they will often postpone work or change plans rather than have their tooth cut. When the case is put to them, that to have the porcelain they will have to sacrifice more of the tooth than would have to be cut for a gold filling, they will decide against the inlay. But woe be unto that dentist when the patients find out the truth in the matter, for his advice then becomes a regular boomerang.

Those persons who have heard enough about inlays to ask their dentists for them, will sooner or later learn more about them, and then they will know that their dentist's argument was a subterfuge behind which to hide his inability, and that more tooth structure was cut to prepare for the gold filling than would have been done to properly prepare for an inlay. Several such individuals have become patients of mine, and I don't think they are now working very hard for the other dentists. If you cannot successfully perform a given operation with porcelain, tell your patients so; tell them that you have done very little of that work and don't feel able to undertake that particular case. If you know of any one who is doing porcelain work, tell them that Dr. A. is doing more along that line than you are, and that he might be able to do it for them. Your patients will respect your honesty.

If you are a beginner in the work, and don't feel that you have had experience enough to undertake some difficult case, take an impression of the tooth, select the corresponding tooth, and your impression being your guide, shape a cavity just as the cavity you want to fill will be, or if you can carry the shape of the cavity in your eye, dispense with the impression. Start the separation for working space, and by the time this is accomplished you will have found time to make several matrices and inlays for the dummy tooth, and in this way gain experience for this particular cavity that will enable you to successfully perform the operation for your patient.

To-day you can procure everything in the way of materials and appliances that you could ask for. The item of time is an important factor in all the affairs of to-day. The furnaces of the present have cut down the time consumed in baking porcelain so that we can accomplish at one sitting for the patient what was formerly divided between two sittings. This saving of time has brought porcelain work to the point where any one who is doing fifty percent of his filling with porcelain, as between gold and porcelain, will find that he is accomplishing the porcelain fillings in less time than it takes him to put in gold fillings. There are three things in favor of taking up this work—less time, easier work and more pay.

First, I will tell you what you can do with porcelain. You can restore to full contour and usefulness broken-down teeth that would otherwise be condemned to crowning. Many a tooth which is so far gone that there is not tooth substance enough left in which retention can be cut to anchor a gold or amalgam filling, and which would have to be crowned, can be restored with porcelain. Instead of further weakening by trying to get anchorage you will strengthen, for all the cutting necessary is to remove all decay, the under-cuts, if any, can be filled up with cement, and a porcelain filling, adapted to all parts of the cavity as closely as at the margins, and cemented to place under pressure, makes that tooth practically as strong as it was originally.

You can take those teeth that remain sensitive to thermal changes after being filled with a gold or amalgam filling, not enough to endanger the pulp, but just enough to be a constant annoyance to the patients, who speak to you about it every time they see you, and if you will remove the metal fillings and replace with porcelain their troubles will be over, and they will be remarking how comfortable those teeth are now—that they would not know they had them. Porcelain is the best non-conductor of thermal changes and the best insulator against electric or galvanic action of any material we have ever used, and it restores a tooth to a practically normal condition.

You can take those teeth where decay has been so extensive as to almost make an exposure in its removal, where with all the protection you can give by cavity lining you are afraid the pulp will die under a cement filling, to say nothing of gold or amalgam, and if you will make a porcelain filling, carrying the material to the full depth of the cavity, so that there will be but a film of cement between filling and dentin, you can assure your patient that there is little likelihood of those teeth ever giving any trouble.

You can take a mouth that is a disfirgurement through number of gold fillings that are in sight, and by replacing them with porcelain you can so alter the appearance that at conversational range no one would suspect there were any fillings in the teeth, and they will become a feature of beauty instead of unsightliness.

You can permanently fill teeth with porcelain for the young, the middle-aged, and the old, the nervous, the sickly, and all those with whom heretofore the best you could do was to temporize, because the cavity preparation is so simple and easily accomplished that any of the above-named will allow you to do all that is necessary to properly prepare the cavity.

What to do with porcelain.. First of all have plenty of working space. You cannot put in flat porcelain fillings; you must have room for the inlay to start into the cavity. In the majority of cases you will need no more than is necessary to put in a properly-contoured gold filling. Do not try to get this space at the time of the sitting with a separator, because the latter will handicap you in the completion of the operation. Get working space before the time of the appointment. Having space, you will contour properly and will restore contact points superior to anything you have ever accomplished with cement, amalgam or gold. Your patients will appreciate the changed condition brought to their mouths by having this working space, and they or you will not consider it time wasted.

Through this necessity is going to come a great benefit to the laity and also to the operator. The operator seeing the superior results he is obtaining will separate for all materials he may be using. This will bring him to working up to an ideal standard, and when one is working toward that he is working at his best, and success is sure to crown one's best efforts.

Always prepare the cavity so that the orifice is larger than the interior. If undercuts come because of the removal of decay, and the enamel walls have any dentin supporting them, fill them with cement and shape the cavity as though there had been no undercuts. Bevel all margins to a knife edge with the outer plane or surface of the tooth, having a seating form for setting purposes. This can be accomplished in the majority of cases by a slight flattening of the floor in a triangular form. A No. 2 round bur run around the interior will change a saucer-shapped cavity to a flattened floor, and prevent the inlay from slipping and getting out of place when the cement is in the cavity. It is very discouraging after setting an inlay to find on cleaning away the excess cement that one margin is depressed and the opposite correspondingly raised. A seating form for setting purposes will prevent this. Use gem and Arkansas stones in preparing a cavity wherever it is possible to do so. They will remove decay and shape a cavity with much less pain to the patient than burs.

Always burnish your matrix directly upon the tooth. Taking an impression and making a model, to say the least, is working once removed from the original, and no one would expect to accomplish as good results upon a model three or four times removed as he would upon the original, and there is the same percentage of difference between working upon the tooth and upon a model. Besides, it takes so much more time as to make it impracticable for the busy man, it being out of the question to prepare a cavity, make and set an inlay at one sitting.

Always use platinum 1-1000 of an inch in thickness for your matrix. The best results, everthing considered, will be accomplished with this material and that gauge. Gold would prevent the use of high-fusing bodies, and up to the present time the best all-round results and artistic effects can be accomplished with high-fusing bodies.

Do all your porcelain work with two or more bodies that fuse at different degrees of heat, making a foundation of two-thirds of the work in hand of the highest fusing, finishing with the colors you want to use that are lower fusing. This prevents the repeated bakings disturbing the first or foundation. Disintegration or minute bubbling is almost sure to follow a second or third firing. Using bodies of a different degree of fusing will prevent this and give you the maximum strength with the minimum risk.

Always work without the rubber dam. The dam is a source of discomfort to the majority of patients, and if on will dry out the tooth so that you will not be able to select colors that match inlay to tooth. Always look for underlying shades in the tooth in deciding what colors to use in your inlay; taking a facing that you think is a good match for the tooth and then working to that will not give you good results, because you are working to something once removed from the original. Besides, who ever saw a facing that was an exact match for a tooth? In a tooth there are always some underlying colors that are not in the facing. In baking an inlay you can put these colors in their respective places and thus get match of inlay to tooth. Take the shade guide furnished with every outfit of bodies, and holding it to the tooth determine what colors you see and their strength. It is only by comparison that a correct decision can be arrived at. What may seem to you to be a blue effect may not be blue at all, and it is only by holding blue to the tooth that you discover this, and that when gray is held to the tooth the law of compensation is satisfied and that it is gray you want in the inlay.

Always bake your inlay in layers. This will accomplish three things—translucency and life-like appearance, avoidance of shadow, and reducing to the minimum the cement showing through from underneath Bake into the matrix a foundation that practically makes an inlay for the lingual half of the cavity. Upon this foundation bake the colors you have decided on in their respective places. Never mix two or more bodies together to vary the shade of one. You can get any shade of a given color by the thickness of the layer you bake. The shade guide furnished with every outfit gives the color that body will be if you bake the same bulk to a full glaze. Bake in your colors strong enough that when subdued by a mutual color there will be an enamel layer which will give you the color wanted. This gives you translucency, and the porcelain being in strata will break up light absorption and refraction and thus do away with the shadow problem. The same procedure with the etched surface next to the tooth will reduce to the minimum the chance of cement showing through.

Always try in the inlay before stripping off the platinum and see that the contour and contact point are just right, so that if any grinding is necessary it can be done and the inlay then be returned to the furnace and glazed again. Never grind any surface of an inlay after setting, other than the occlusal surfaces of molars and bicuspids and the cutting edge of the anterior teeth. The glazed surface of porcelain will not retain food deposits or glutinous plaques, and consequently is a protection to surrounding and approximating tooth surfaces.

Always dip matrix and inlay in water if you are bothered in stripping off the platinum, and you will find it will come off easily. This shows that a joint so tight as porcelain baked upon platinum is pervious to water. I will let you draw your own inferences.

Always etch the reverse side of the inlay with hydrofluoric acid. This will leave a roughened surface that cement will adhere to very tightly and still not remove surface enough to take away from close adaptation. Imbed the inlay in wax to protect the exposed surface, and then a drop or two of hydrofluoric acid left for three to five minutes will etch sufficiently.

Put on the rubber dam wherever it is possible to do so, to set an inlay, as that insures dryness and a reasonable degree of crystallization before moisture bathes the tooth. Always secure pressure, either by wedge or ligature, and leave under dam and pressure for thirty minutes. Pressure will give a better quality of cement, and will prevent the expansion of crystallization from making a perceptible joint of what was a good-fitting insay.

Always wait until the dam is off and moisture has bathed the tooth before removing excess cement. It will then loosen easily and there will be no danger of dislodging the inlay. Always leave the slight line of cement at the joint to wear away, which will be in a few days. Mix the cement as near the color of the tooth as possible, so that the cement at joint will not be conspicuous.

Always show the inlay to the patient set and wet by the saliva. At no time will it ever look better, and first impressions are often lasting. Say that the drying out of the tooth under the rubber dam makes it several shades lighter, that it will be several hours before the tooth is back to color again, and that the inlay will look better a week hence than it does the first day. Tell the patient that he will always be able to see the inlay, that it is like the puzzle pictures in the papers—when you have once solved the picture you can always see it. Others at close conversational range will not see the inlay nor imagine there is a filling in the tooth.—*Digest.*

